# Case Report: First case of Triple A syndrome diagnosed in French Guiana: clinical presentation, molecular confirmation, and literature review

**DOI:** 10.3389/fped.2026.1906922

**Published:** 2026-09-23

**Authors:** Mody Diop, Telcide Roosler Billy, Narcisse Elenga

**Affiliations:** 1 Specialized Clinical Genetics Consultation, Women-Child Division, CH Andrée Rosemon, Cayenne, French Guiana; 2 Pediatric Endocrinology Department, Women-Child Division, CHU de Guyane, Cayenne, French Guiana; 3 Pediatrics Department, Women-Child Division, CHU de Guyane, Cayenne, French Guiana

**Keywords:** AAAS gene, achalasia, congenital alacrima, primary adrenal insufficiency, Triple A syndrome

## Abstract

Triple A syndrome (OMIM #231550) is a rare autosomal recessive disorder defined by the triad of alacrima, adrenal insufficiency, and achalasia. Around 100 to 150 cases have been reported in the world literature since 1978. We report the first case diagnosed in French Guiana, in a 5-year-old boy who presented with severe hypoglycemic episodes. Exome sequencing identified a homozygous pathogenic variant in the AAAS gene (NM_015665.6:c.43C > A, p.Gln15Lys — ACMG Class 5). Family testing confirmed heterozygous carrier status in both parents, while the younger brother was found to be homozygous wild-type and unaffected. The clinical course under hydrocortisone replacement has been favorable. A literature review and comparative analysis are presented to place this case in context and to highlight the phenotypic and molecular variability of the syndrome.

## Introduction

Triple A syndrome (OMIM #231550) is a rare autosomal recessive disorder. Its name refers to the three findings that make up the classic triad: alacrima (absence of tears), achalasia (swallowing difficulty due to esophageal dysfunction), and adrenal insufficiency (addisonism) (1). It is thought to affect around 1 person per million, without any clearly established sex or ethnic predominance, and about 100 to 150 cases have been described worldwide since it was first reported (2).

The gene responsible, AAAS, sits on chromosome 12q13 and encodes ALADIN, a protein involved in nucleocytoplasmic transport (3, 4). Over 65 variants have now been described. Some cluster geographically, pointing to founder effects in certain populations, but overall there is no clear relationship between a given variant and the severity or pattern of disease (5).

Patients differ considerably from one another, and the three cardinal signs rarely appear at the same time. Alacrima tends to come first, often from birth, though it is easy to miss. Adrenal insufficiency, present in around 85% of patients, can announce itself abruptly through severe, potentially life-threatening hypoglycemia. Achalasia usually comes later, sometimes years afterward. Given how rare and variable the presentation is, Triple A syndrome is often diagnosed late, occasionally after several years of unexplained symptoms.

We describe here the first case of Triple A syndrome diagnosed in French Guiana that has been confirmed both clinically and molecularly. It is a reminder to consider this diagnosis in any child with non-autoimmune adrenal insufficiency and congenital alacrima, and it adds to a body of literature on this rare disease that remains thin for French overseas territories. A review of the literature and a comparative analysis follow the case description.

## Case presentation

### Reason for consultation and history

A 5-year-old boy, born and living in Kourou (French Guiana), was hospitalized at CHU de Guyane in October 2025 for recurrent episodes of malaise. His history included a severe hypoglycemic episode (0.2 g/L) with seizures in May 2025. His parents had noticed progressive skin hyperpigmentation since early 2025, as well as a near-total absence of tears since birth that had not previously been reported to medical staff. Psychomotor development had been normal. There was no history of parental consanguinity.

### Clinical examination

On admission, the child showed marked hyperpigmentation over the whole body. Vital signs were normal, and the abdomen was soft with no hepatosplenomegaly. His parents reported intermittent motor fatigability. Alacrima was confirmed on questioning. External genitalia were normal. Anthropometry: weight 22.5 kg, height 114 cm, BMI 17.3 kg/m^2^ (90th percentile).

### Biochemical and hormonal work-up

Routine blood tests were normal. Hormonal testing showed a very low morning cortisol (0.2 µg/dL), markedly elevated ACTH (1726ng/L), 17-OH-progesterone below 0.10 ng/mL, and normal IGF-1. A full autoimmune panel (anti-21-hydroxylase, anti-TPO, anti-thyroglobulin, anti-transglutaminase antibodies) was negative, arguing against an autoimmune cause.

### Imaging

Abdominal CT showed bilateral adrenal atrophy without mass or calcification, consistent with chronic, non-autoimmune adrenal insufficiency. Chest x-ray was normal.

### Initial management

Hydrocortisone replacement was started urgently at 12 mg/m^2^/day, given in three doses (5 mg/2.5 mg/2.5 mg). A stress-dosing plan was set up, with dose doubling or tripling for fever and an intramuscular option for vomiting. Parental education began during the hospital stay, and the family's adherence has been excellent.

### Genetic confirmation and family investigation

The combination of non-autoimmune primary adrenal insufficiency, congenital alacrima, and bilateral adrenal atrophy raised suspicion of Triple A syndrome. Solo exome sequencing (Eurofins Biomnis Lyon) identified a homozygous pathogenic variant in the AAAS gene: NM_015665.6:c.43C > A, p.(Gln15Lys), ACMG Class 5, subsequently confirmed by Sanger sequencing.

Family testing showed that both parents (father, 33 years; mother, 34 years; not related to each other) were heterozygous carriers of the same variant. The younger brother was also tested and found to be homozygous wild-type — unaffected and not a carrier. The family pedigree is shown in Figure 1.

**Figure 1:**
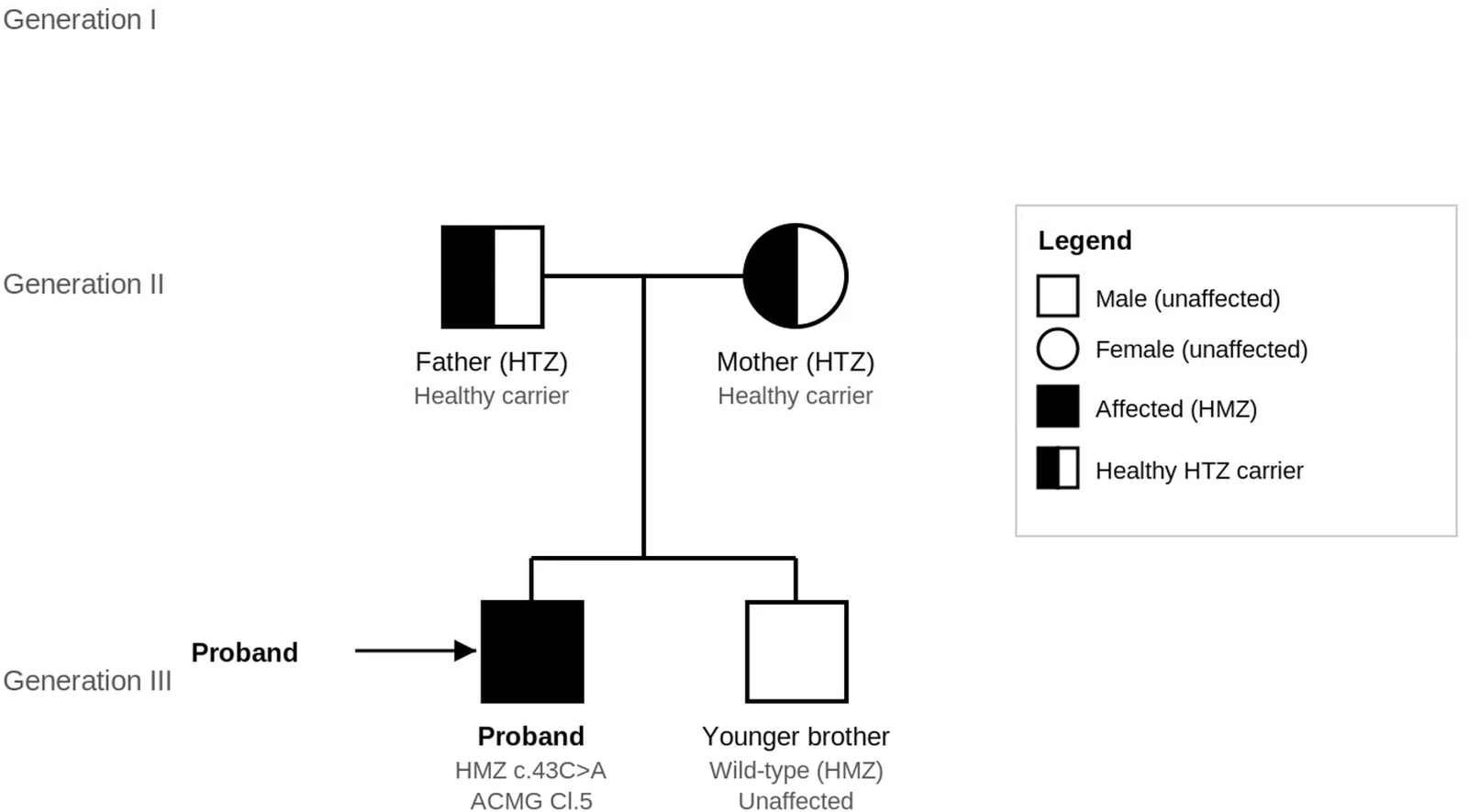
Anonymized family pedigree. Square=male, circle=female. Fully filled symbol=affected (homozygous, index case). Half-filled symbol=healthy heterozygous carrier. The parents are alive, unrelated, and both healthy heterozygous carriers. The younger brother is homozygous wild-type and unaffected.

### Course and follow-up

Since starting hydrocortisone, the child's clinical course has been very favorable: fatigability has resolved, skin pigmentation has progressively normalized, there have been no further hypoglycemic episodes, and growth has been satisfactory, with a 3 cm gain over 5 months (height 117 cm, weight 23 kg, body surface area 0.87 m^2^). Alacrima has persisted without conjunctival inflammation. No sign of achalasia has been found so far. Mineralocorticoid function remains normal. Multidisciplinary follow-up has been organized, including ophthalmology (Schirmer test), gastroenterology, pediatric endocrinology, and neuropediatrics.

## Literature review

### Epidemiology and reported cases worldwide

Since 1978, roughly 100 to 150 cases of Triple A syndrome have been reported, mostly as case reports or small family series (1, 2). The syndrome has been described on every continent, without a clear ethnic predominance. A 2024 systematic review found a mean age at diagnosis of around 5 years (6). The true number of cases is probably underestimated, largely because of frequent underdiagnosis when the triad is incomplete.

### Clinical spectrum and phenotypic variability

The classic triad is complete in roughly two-thirds of cases. Alacrima is the earliest and most consistent sign (7). Adrenal insufficiency is glucocorticoid-predominant in most patients, with mineralocorticoid function preserved in around 85% (5). Achalasia can appear later, which justifies prolonged gastroenterological follow-up (8). Neurological features (autonomic dysfunction, peripheral neuropathy, pyramidal signs) can also occur, giving rise to the terms “4A” or “5A syndrome” (3). Phenotypic variability, including within the same family, is a recognized feature of the disease and appears independent of genotype (5, 9).

### Molecular diversity of the AAAS gene

More than 65 pathogenic variants have been identified in the AAAS gene, with considerable molecular heterogeneity and some well-documented geographic founder effects. The c.1331 + 1G > A variant predominates in North Africa (9), while p.Ser263Pro is common in Eastern Europe (3). The c.43C > A p.(Gln15Lys) variant identified in our patient has mainly been reported in South Asian populations (5). No genotype-phenotype correlation has been established to date.

### Management and follow-up

Treatment relies on glucocorticoid replacement with hydrocortisone (8–15 mg/m^2^/day in three divided doses), adjusted for periods of stress, along with artificial tears for alacrima and endoscopic (pneumatic dilation) or surgical (Heller myotomy) management of achalasia when it occurs (8). Long-term multidisciplinary follow-up is essential to detect neurological complications (7).

## Discussion

As far as we can tell, this is the first case of Triple A syndrome in French Guiana to be confirmed both clinically and at the molecular level. Setting it alongside the published series (Table 1, Figures 2–6) helps situate our observation within the broader literature and points to a few clinical, molecular, and organizational lessons.

**Table 1:** Comparison of the main published series and cases of triple A syndrome with the present case (highlighted).

Author (year)	Country	N	Sex	Age at dx	Alacrima	Addison	Achalasia	Neuro	AAAS variant
Allgrove et al. (1978)	United Kingdom	4	2M/2F	Childhood	Yes	Yes	Yes	NR	NR
Houlden et al. (2002)	United Kingdom	20	M/F	3–25 y	Yes	Yes	Yes	Yes	Multiple
Kallabi et al. (2016)	Tunisia	26	12M/14F	1–10 y	26/26	26/26	23/26	Yes	c.1331 + 1G > A
Kurnaz et al. (2018)	Turkey	6	5M/1F	3–7.5 y	6/6	6/6	4/6	Yes	Multiple
Patt et al. (2017)	India	Review	M/F	Variable	Yes	Yes	Yes	Variable	>65 variants
Gaiani et al. (2020)	Italy	2	0M/2F	6–8 y	2/2	1/2	2/2	No	c.1024C > T
Shah et al. (2017)	Pakistan	2	0M/2F	6–12 y	2/2	2/2	2/2	NR	NR
Belmokhtar et al. (2023)	Morocco	10	8M/2F	2–11 y	Yes	Yes	Yes	NR	c.1331 + 1G > A
Cehic et al. (2024)	Serbia	1	1M	2 mo	Yes	Yes	Yes	Yes	c.769G > A
Present case (2025)	French Guiana	1	1M	5 y	Yes	Yes	No*	No	c.43C > A (HMZ)

NR = not reported; HMZ=homozygous; * achalasia not identified at this stage of follow-up.

**Figure 2:**
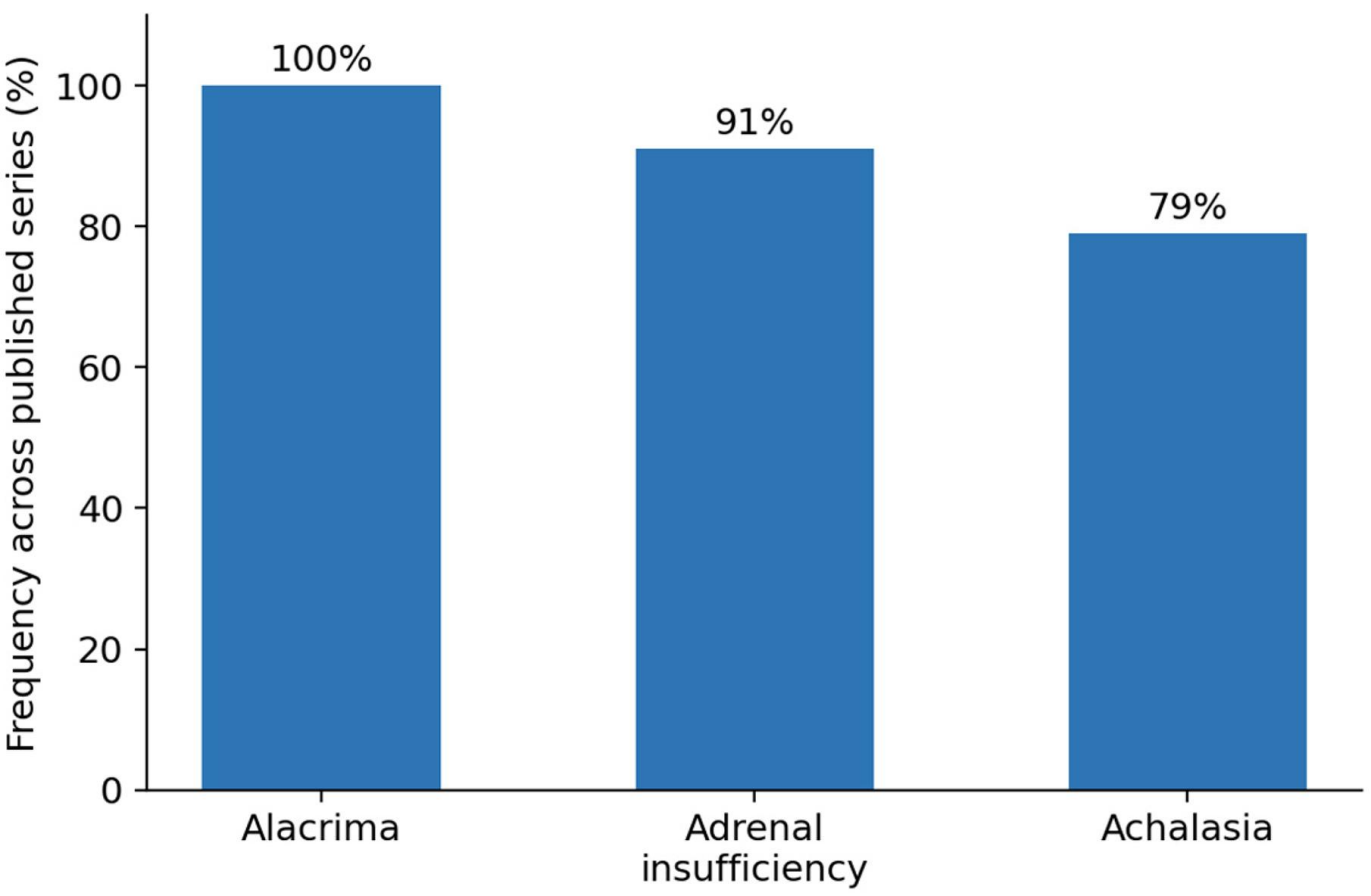
Frequency of the three components of the triple A triad across published series. Alacrima is present in virtually all cases (100%), followed by adrenal insufficiency (91%) and achalasia (79%).

**Figure 3:**
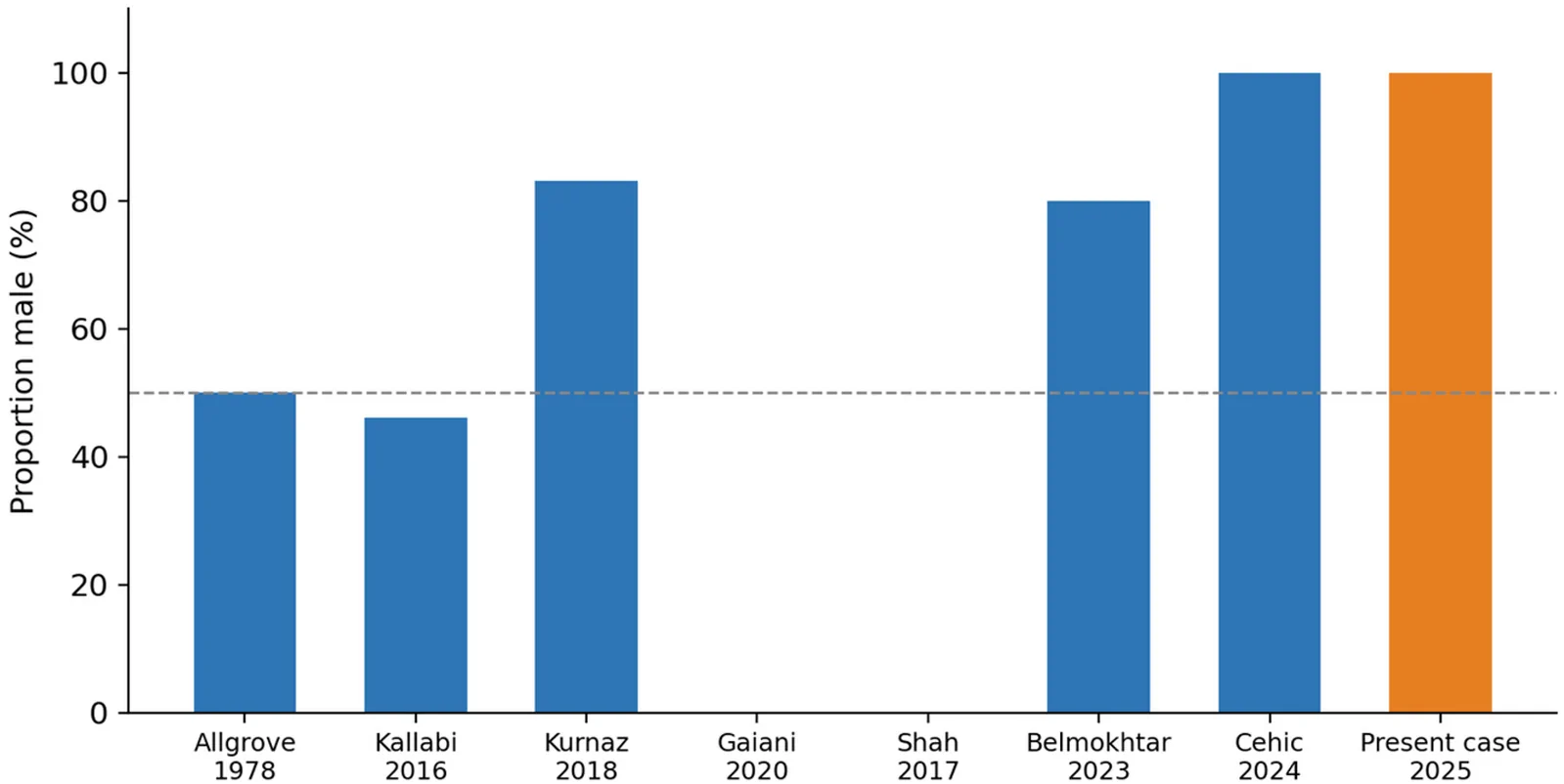
Sex ratio by published series. A slight male predominance is seen in most series, though it varies with study size and design.

**Figure 4:**
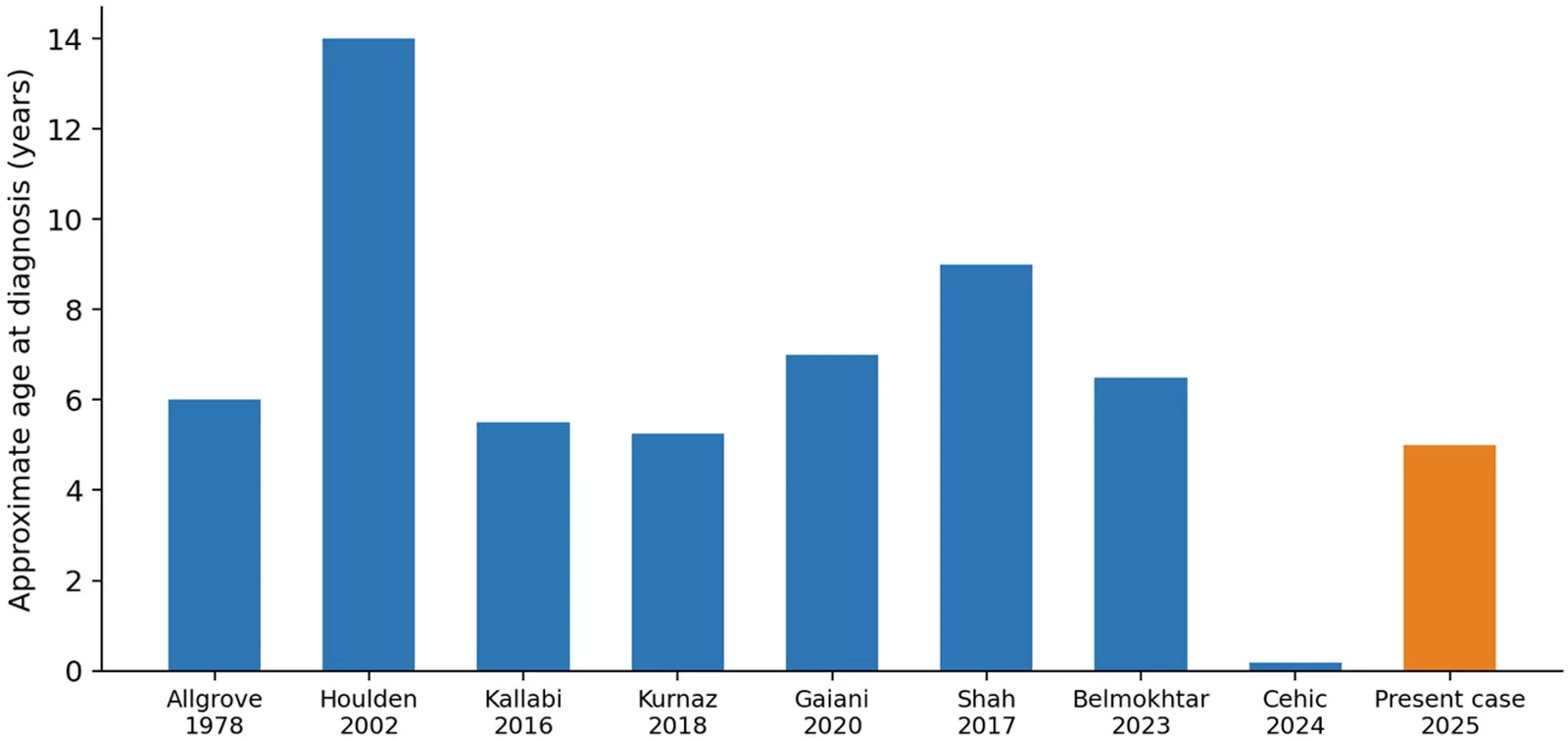
Approximate age at diagnosis by series (midpoint of reported ranges, for illustration). The present case (orange, age 5) falls close to the median reported in the world literature.

**Figure 5:**
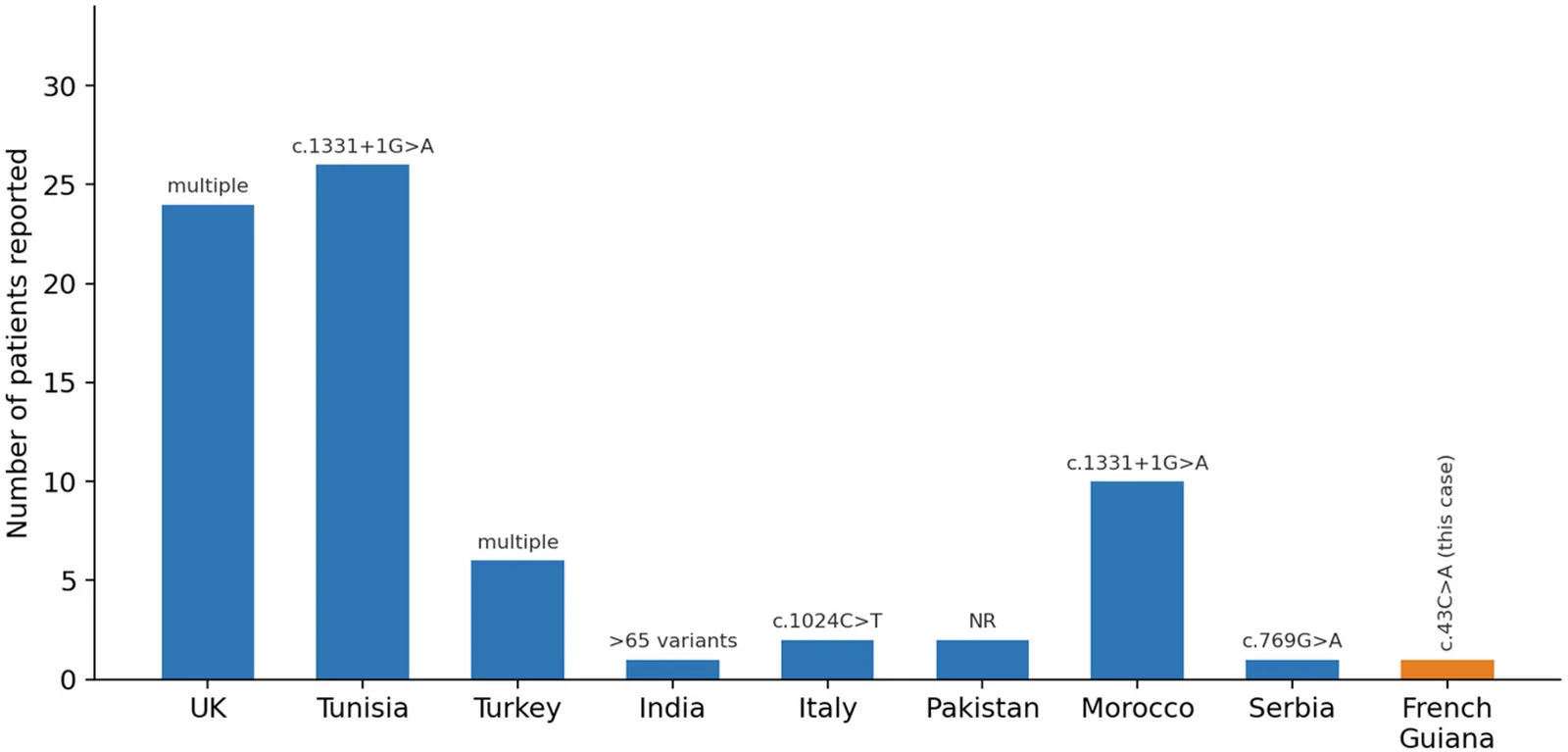
Number of reported patients and associated aAAS variant(s) by country of origin. The North African founder variant c.1331 + 1G > A predominates in several series. The c.43C > A variant identified in the present case (orange) had not previously been reported in this geographic context.

**Figure 6:**
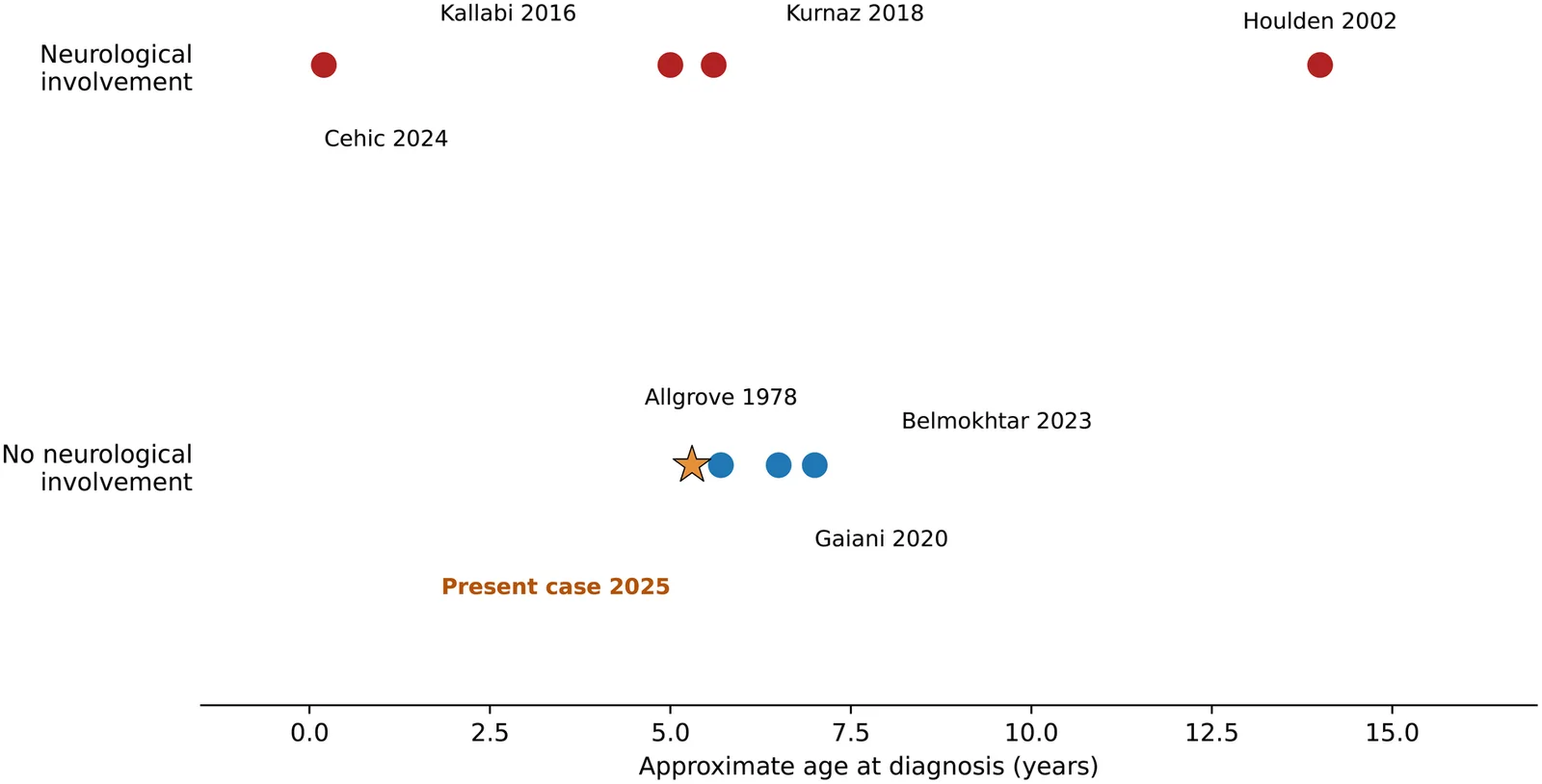
Relationship between approximate age at diagnosis and neurological involvement across published series. Neurological involvement (red points) is seen mainly in patients diagnosed later in life. The present case (orange star) shows no neurological involvement at age 5.

### Age at diagnosis: a presentation consistent with the literature

Age at diagnosis varies enormously across the published series (Figure 4) — from 2 months in the case reported by Cehic et al. in 2024 to as late as 25 years in the Houlden et al. cohort from 2002 (2, 6). Our patient, diagnosed at age 5, sits close to the reported median. Even though alacrima is present from birth, it seems the diagnosis is rarely reached before something more alarming happens, which in our patient's case was a hypoglycemic seizure.

### Male predominance varies across series

Figure 3 shows a slight male predominance in most series (sex ratio around 1.3). The Tunisian series (12M/14F out of 26 patients), however, is close to balanced (5, 10), suggesting this predominance may partly reflect recruitment bias rather than a true biological difference.

### Alacrima: a universal but underrecognized sign

Figure 2 is unambiguous: alacrima is present in essentially all cases when specifically looked for. Yet in our patient, as in most published cases, this sign went unrecognized for years. Alacrima is arguably the most reliable early warning sign of Triple A syndrome, and raising awareness of it among parents and frontline clinicians matters.

### Adrenal insufficiency: variable presentation

Adrenal insufficiency was present in 91% of the cases reviewed (Figure 2). Our patient's ACTH of 1726ng/L together with a markedly low cortisol of 0.2 µg/dL is among the more severe values reported in pediatric series. The preserved mineralocorticoid function, consistent with the roughly 85% preservation rate reported in the literature, meant fludrocortisone was not needed (5).

### Achalasia: the least constant component

Achalasia was absent in about 21% of the cases reviewed (Figure 2), which is why incomplete presentations are sometimes described as “double A syndrome” (5, 8). Its absence at diagnosis does not rule out Triple A syndrome when the other two components are present and molecular confirmation has been obtained. Prolonged gastroenterological monitoring remains warranted.

### Neurological involvement: uncommon in early pediatric presentations

Figure 6 confirms that neurological manifestations are essentially absent in early pediatric series but appear in series that include older patients (2, 9). The lack of neurological involvement in our 5-year-old patient is expected at this stage but still justifies regular long-term neuropediatric follow-up.

### Molecular diversity: our variant in a global context

Figure 5 shows the geographic distribution of AAAS variants. The c.43C > A p.(Gln15Lys) variant, absent from North African and European series, has mainly been reported in South Asian populations (5). Its occurrence at the homozygous state in a child from French Guiana, born to unrelated heterozygous parents, is a notable finding, and supports the use of exome sequencing rather than targeted testing in genetically diverse populations such as that of French Guiana.

### Family segregation and genetic counseling

Family testing confirmed classic autosomal recessive transmission (Figure 1): two heterozygous parents, one affected homozygous child, and a younger brother who is homozygous wild-type and unaffected. The recurrence risk for future pregnancies in this couple is estimated at 25%. Genetic counseling has been offered to the family.

### Management and outlook

Physiological-dose hydrocortisone replacement led to a rapid and substantial clinical improvement. Because mineralocorticoid function was preserved, fludrocortisone was not required (8). Management of alacrima relies on artificial tears and regular ophthalmological follow-up to prevent corneal complications. Prolonged multidisciplinary follow-up remains necessary given the risk of later achalasia and delayed neurological complications (7).

### Implications for French Guiana

French Guiana is a territory with substantial ethnic diversity and limited local access to rare-disease care pathways. This case shows that an accurate diagnosis, including molecular confirmation and family investigation, is achievable through collaboration between local clinical genetics and specialized metropolitan laboratories. It also supports the case for strengthening rare-disease care pathways in French overseas territories.

## Conclusion

Diagnosing Triple A syndrome means recognizing a triad that is often incomplete, and confirming it through molecular analysis of AAAS. Looking across the published series (Figures 2–6), a few things stand out: alacrima is an early sign that gets missed far too often, the triad does not need to be complete for the diagnosis to hold, and the molecular spectrum of the disease is broader than any single population can capture. This first documented case in French Guiana is a reminder that the condition can affect children regardless of background. Starting glucocorticoid replacement early can prevent life-threatening complications, and given the risk of achalasia or neurological problems emerging later, follow-up needs to continue well beyond the initial diagnosis. We hope this case adds something useful to what is known about the geographic and phenotypic range of Triple A syndrome, and makes the case, once again, for stronger rare-disease pathways in French overseas territories.

## Data Availability

The original contributions presented in the study are included in the article/Supplementary Material, further inquiries can be directed to the corresponding author.
