# Supplementary material for: Case Report: First case of Triple A syndrome diagnosed in French Guiana: clinical presentation, molecular confirmation, and literature review
Source: Front Pediatr. 2026 Sep 23;14:1906922. doi: 10.3389/fped.2026.1906922 (PMC13642778; doi:10.3389/fped.2026.1906922)
Supplement: Supplementary file 1 [file Table1.docx]

**Supplementary Table 1**

Summary of published case series of Triple A syndrome (1978–2025), compared with the present case (highlighted in yellow).

NR: not reported; HMZ: homozygous; HTZ: heterozygous; Cl.: ACMG class; dx: diagnosis; mo: months; yrs: years.

| **Reference** | **Country** | **N** | **Age at dx** | **Alacrima** | **Adrenal insuff.** | **Achalasia** | **Neurology** | **AAAS variant** |
| --- | --- | --- | --- | --- | --- | --- | --- | --- |
| Allgrove et al., 1978 | UK | 4 | Childhood | 100% | 100% | 100% | NR | Not identified |
| Huebner et al., 1999 | Multi-country | 11 | 2–18 yrs | 100% | 91% | 82% | 55% | Multiple |
| Dumic et al., 2012 | Croatia | 3 | 3–14 yrs | 100% | 100% | 67% | 33% | c.1331+1G>A HMZ |
| Kallabi et al., 2016 | Tunisia | 26 | 1–25 yrs | 100% | 100% | 85% | 42% | c.1331+1G>A (founder) |
| Shah et al., 2017 | Pakistan | 1 | 8 yrs | Yes | Yes | No | Yes | Not identified |
| Patt et al., 2017 | India | 12 | 4–22 yrs | 100% | 100% | 75% | 58% | Multiple |
| Gaiani et al., 2020 | Italy | 3 | 2–35 yrs | 100% | 100% | 100% | 67% | c.1331+1G>A HTZ |
| Belmokhtar et al., 2023 | Morocco | 8 | 2 mo–18 yrs | 100% | 100% | 88% | 50% | c.1331+1G>A HMZ |
| Cehic et al., 2024 | Bosnia | 1 | 2 months | Yes | Yes | No | Yes | c.773G>A HMZ |
| Dumic et al., 2012 (follow-up) | Croatia | 3 | 12 yrs (follow-up) | 100% | 100% | 100% | 100% | c.1331+1G>A HMZ |
| **Diop et al., 2025** | **French Guiana** | **1** | **5 yrs** | **Yes** | **Yes** | **No (at this stage)** | **Absent** | **c.43C>A p.(Gln15Lys) HMZ Cl.5** |
